# Artificial Intelligence Informed Hydrogel Biomaterials in Additive Manufacturing

**DOI:** 10.3390/gels11120981

**Published:** 2025-12-06

**Authors:** Zhizhou Zhang, Zach Z. Tao, Ruiling Du, Runxin Huo, Xiangrui Zheng

**Affiliations:** 1 School of Engineering, The University of Manchester, Manchester M13 9PL, UK; 2 Faculty of Engineering, University College London, London WC1E 6BT, UK; 3 School of Materials Science and Engineering, Central South University, Changsha 410083, China; 4 Department of Biochemistry, Yong Loo Lin School of Medicine, National University of Singapore, Singapore 117596, Singapore

**Keywords:** hydrogel, biomaterials, machine learning, additive manufacturing, material design

## Abstract

Hydrogel additive manufacturing underpins soft tissue models, biointerfaces, and soft robotics. The coupled choices of formulation, rheology, and process conditions limit the progress. This review maps how artificial intelligence links composition to printability across direct ink writing, inkjet, vat photopolymerization, and laser-induced forward transfer, and how vision-guided control improves fidelity and viability during printing. Interpretable predictors connect routine rheology to strand stability, data-driven classifiers chart droplet regimes, and optical dose models with learning enhance voxel accuracy. Polymer informatics, including BigSMILES based representations, supports generative screening of precursors and crosslinkers. Bayesian optimization and active learning reduce experimental burden while honoring biological constraints, and emerging autonomous platforms integrate in situ sensing with rapid iteration. A strategic framework outlines a technological progression from current open-loop data gathering toward real-time closed-loop correction and ultimately predictive fault prevention through digital twins. The synthesis provides quantitative routes from formulation through process to function, establishing a practical foundation for predictive, reproducible hydrogel manufacturing and application-oriented design.

## 1. Introduction

**Figure 1:**
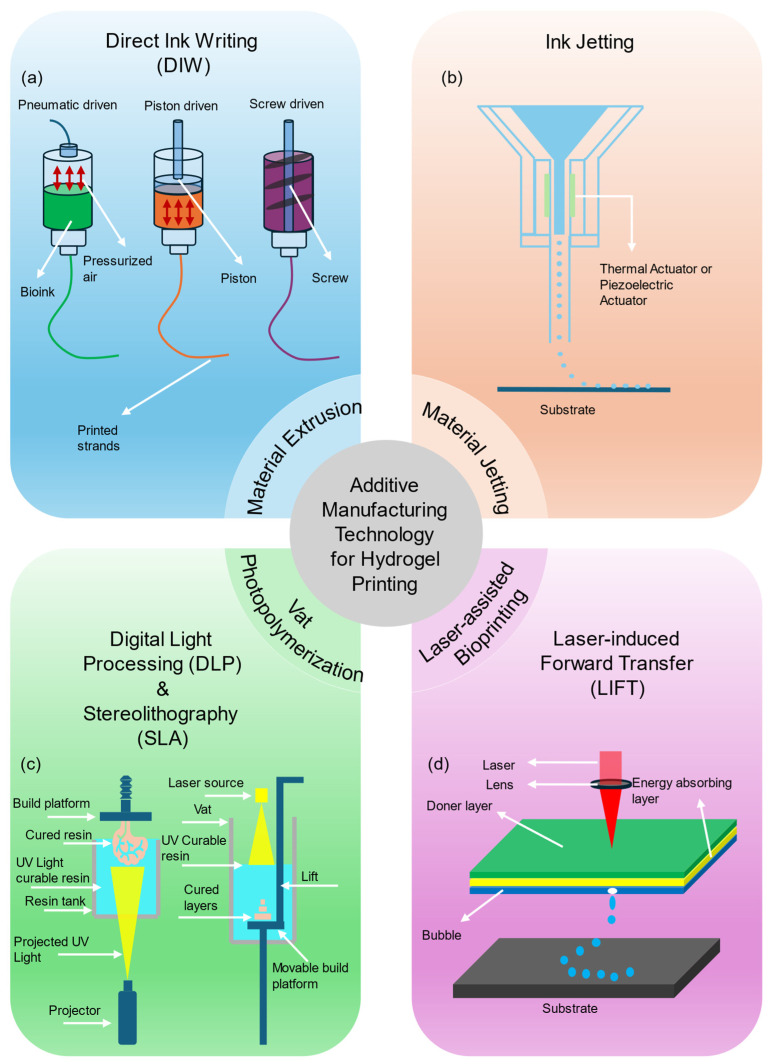
Schematics of hydrogel printing: (**a**) direct ink writing (DIW), (**b**) ink jetting, (**c**) digital light processing (DLP), stereolithography (SLA), (**d**) laser-induced forward transfer (LIFT).

Hydrogel biomaterials supported soft tissue engineering, bioelectronics, and soft robotics but their translation through additive manufacturing remained constrained by complex chemistries, non-Newtonian flow, and sensitivity to processing histories [1,2]. Reviews of printability and rheology summarized how viscosity in Pa·s, storage modulus in Pa, and recovery kinetics governed extrusion fidelity and shape retention, and they urged measurable criteria for print success across natural and synthetic systems [2,3,4]. In parallel, polymer informatics matured through standardized representations and accessible datasets, which positioned artificial intelligence to connect formulation, rheology, and printing outcomes through data-grounded models [1,5,6]. The field converged on measurable structure–process–property relations and on digital assets that enabled predictive workflows for hydrogel in additive manufacturing. Figure 1 illustrated four hydrogel additive manufacturing modalities, showing direct ink writing with pneumatic, piston, and screw actuation, ink jetting with thermal or piezoelectric actuation, vat photopolymerization through digital light processing and stereolithography, and laser-induced forward transfer deposition onto a substrate.

Table 1 summarized hydrogel additive manufacturing methods by pairing each technology with its printing type and the governing process parameters, including rheology and nozzle settings for direct ink writing, fluid properties and actuation controls for ink jetting, photochemistry and exposure conditions for digital light processing and stereolithography, and laser energy and donor film characteristics for laser-induced forward transfer.

Prior work already demonstrated that artificial intelligence learned printability from routine rheology and reduced empirical tuning. An interpretable pipeline trained on 180 formulations identified 13 rheological descriptors that consistently separated printable from non-printable inks across chemistries [38]. A complementary database of 150 printed hydrogel established quantitative mappings from rheological indices to filament formation and part fidelity, enabling laboratories to estimate pressure and speed windows from standardized oscillatory and flow tests [39]. These studies aligned with extrusion theory where yield stress, flow index, and shear rate controlled bead continuity in cylindrical nozzles [3,4]. Datasets at the scale of 150 to 180 cases delivered general rules that allowed prediction of print success from rheology alone and reduced trial counts before fabrication.

Artificial intelligence also enabled optimization under biological constraints. A combined neural network and Bayesian optimization workflow predicted cell survival from nozzle diameter, pressure, and speed for gelatin and alginate systems and reported coefficient of determination 0.71 with classification accuracy 0.86 on viability labels, which supported guided search of operating points rather than exhaustive grids [40]. Mechanistic modeling in physical review work quantified wall shear stress and extensional stress histories inside and at the exit of nozzles and produced predictive links between pressure drop, nozzle geometry, and cellular loading [41,42]. Together, these results suggested that viability-constrained optimization could respect stress thresholds in the range of 2 to 5 kPa reported for sensitive cells while maintaining geometric fidelity. Artificial intelligence combined with flow physics enabled viable operating maps with quantitative accuracy on survival and reduced the number of printed trials needed for hydrogel constructs.

Integration of computer vision with learning improved geometric accuracy during printing. A vision-based compensation method compared imaged filaments to commanded paths and adjusted the toolpath to reduce width and area errors in robotic bioprinting, demonstrating practical closed-loop correction during multi-layer deposition [43]. Image-informed learning also connected microstructure to mechanics, where a data-driven pipeline predicted elastic modulus from scanning electron microscopy images within the correct order of magnitude and exposed texture features that reflected pore size and connectivity [44]. Reviews of inkjet and lithography strategies emphasized that high-resolution hydrogel patterning depended on waveform control, viscosity, and photochemical kinetics and that artificial intelligence could accelerate design across droplet-scale and voxel-scale regimes with typical feature sizes near 10 to 50 µm [45,46]. Vision and imaging enriched monitoring and prediction, enabling geometry control during deposition and non-destructive inference of stiffness from microstructure.

Progress in digital chemistry and polymer informatics supported generative and inverse design for hydrogel precursors and networks. BigSMILES provided a machine-readable line notation for stochastic macromolecules and improved curation of repeat unit distributions and branching [47]. Generative BigSMILES extended this notation for ensemble-aware design and supplied the metadata needed for enumeration and ranking in inverse workflows [48]. Community platforms such as Polymer Genome delivered near instantaneous property predictions after curation of training data, while PoLyInfo documented machine-readable schemas that captured structures, properties, and measurement conditions and RadonPy generated synthetic property labels through automated molecular dynamics for more than 1000 amorphous polymers [46,49,50]. Standardized representations and large curated resources enabled artificial intelligence to propose and evaluate hydrogel building blocks while preserving provenance and uncertainty.

Autonomous experimentation and self-driving laboratories promised to compress design cycles for hydrogel inks and printing recipes. Reviews of self-driving laboratories described closed-loop architectures that combined robotics, active learning, and in situ sensing, and reported large gains in search efficiency relative to manual workflows [51]. Demonstrations in polymer processing showed that autonomous platforms could discover processing pathways that achieved conductivities near 2.0 × 10^6^ S·m^−1^ in thin films, illustrating rapid exploration of coupled composition and process spaces that had direct analogs in hydrogel ink preparation and curing [52]. Automation coupled to active learning offered order of magnitude reductions in iteration time for formulation and process optimization relevant to hydrogel.

Despite this momentum, existing reviews and printability models remained fragmented and left several practical questions unresolved for hydrogel additive manufacturing. Reviews centered on hydrogel rheology and shape fidelity typically treated artificial intelligence only as a future opportunity and did not provide quantitative guidance on how learning algorithms should be coupled to formulation, monitoring, and control in routine workflows [1,2]. Conversely, broader surveys of artificial intelligence in additive manufacturing largely focused on metals or dry polymers, with limited attention to hydrated, cell-laden systems and without reporting rheology windows, viability envelopes, or open datasets that were directly reusable for hydrogel inks [45]. Many printability models further targeted single chemistries, proprietary printers, or narrow operating ranges, which hindered transfer of rules between laboratories and obscured how uncertainty should be handled when extrapolating to new gels. Community analyses highlighted data sparsity, heterogeneous measurement protocols, and representation ambiguity as core obstacles for reliable generalization in polymers and gels [1,2], and uncertainty quantification remained underused in hydrogel applications even though recent studies in materials modeling recommended calibrated prediction intervals, ensemble strategies, and physics-guided priors to flag out of distribution conditions during optimization and control [53,54]. Application-focused reviews of bioinks emphasized unresolved tradeoffs between printability and function and called for shared benchmarks that reported viscosity, modulus, and viability in SI units under well-documented test conditions. Together, these gaps motivated a consolidated review that not only summarized the literature but also defined quantitative, transferable problem statements for artificial intelligence in hydrogel additive manufacturing. Standardized datasets with clear metadata and uncertainty were needed to support trustworthy artificial intelligence in hydrogel additive manufacturing.

In response to this gap, this review presented a structured synthesis that opened with an introduction to hydrogel additive manufacturing and a comparative overview of process families, followed by a parameter map that collated governing variables for direct ink writing, ink jetting, digital light processing, stereolithography, and laser-induced forward transfer, then developed sections on artificial intelligence-guided material design, three-dimensional printability prediction, process monitoring and control, case studies, and concluded with an outlook on automation and standards. Its primary contributions lay in unifying rheology to printability relations across chemistries, aggregating quantitative operating windows, curating representative datasets and open resources, and framing active learning and Bayesian optimization as sample efficient routes from formulation to print success, thereby providing reproducible workflows and metrics that supported cross-laboratory transfer and accelerated translation of artificial intelligence enabled hydrogel manufacturing.

## 2. Hydrogel Material Design and 3D Printability by Artificial Intelligence

### 2.1. Artificial Intelligence Design for Hydrogel

Hydrogel design spanned an immense combinatorial space that multiplied chemistry through crosslinking routes and processing parameters, while each experimental iteration required hours to days and substantial consumables. Typical direct ink writing formulations that succeeded in strand retention used zero shear viscosity in the range 10^2^ to 10^4^ Pa·s and yield stress in the range 10 to 10^3^ Pa, with nozzle diameters near 0.1 to 0.4 mm and speeds near 5 to 50 mm·s^−1^, which already defined a high-dimensional search even before accounting for degree of substitution, photoinitiator mass fraction, and solvent ionic strength [38,55]. Lithography-based printing added optical dose and absorption constraints governed by the Jacobs working curve, and interlaboratory studies reported up to 7-fold spreads in light penetration depth and up to 70-fold spreads in critical radiant exposure for nominally identical resins, which underscored the need for data-driven standardization [56,57]. Polymer informatics platforms demonstrated that predictive models could screen millions of candidates in seconds for thermomechanical properties, and recent chemical language models extended this screening to 100 million hypothetical polymers with multi-property inference [6,50]. The size and variability of the hydrogel design space made machine learning an essential accelerator for narrowing feasible regions before costly experiments.

**Figure 2:**
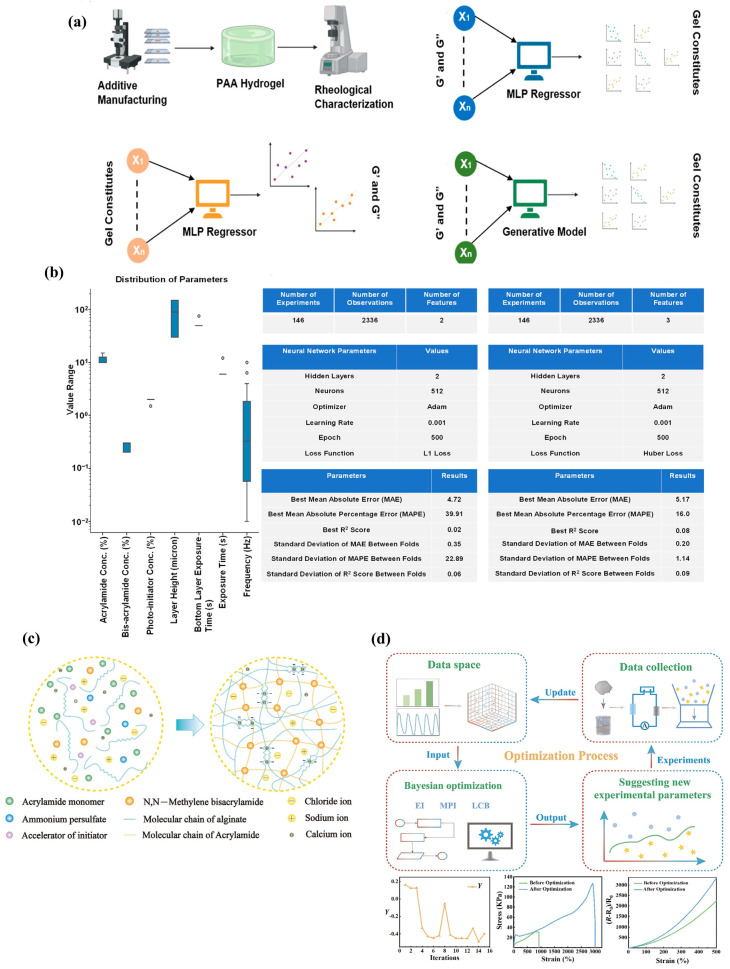
Case studies of AI design for hydrogel. (**a**) The AI model is used to train different components of the hydrogel, conduct the process of testing rheological properties, and finally generate a hydrogel material composition that matches the original data using the generated model [58]. (**b**) Only by using rheological properties can the optimal hyperparameters and results of the hydrogel material be predicted [58]. (**c**) The design principle of interpenetrating double-network hydrogel based on polyacrylamide and alginate [59]. (**d**) Optimizing the Bayesian optimization process of the dual-network hydrogel and the subsequent performance improvement after iterations [59]. Panels (**a**,**b**) adapted with permission from ref. [58], MDPI, CC BY 4.0. Panels (**c**,**d**) adapted with permission from ref. [59], Oxford University Press, CC BY 4.0.

Figure 2a depicted the four-stage workflow that generated rheology data from three three-dimensional printed polyacrylamide hydrogel, trained a multi-layer perceptron to predict storage and loss moduli, attempted inverse prediction, and finally used variational autoencoder and conditional variational autoencoder to generate constituent recipes. Figure 2b summarized the attempt to predict gel components from target moduli, showing variable distributions, best hyperparameters, and outcomes where adding frequency improved mean absolute percentage error but not mean absolute error or R^2^, leading to adoption of generative models instead. Figure 2c depicted the design of a polyacrylamide and alginate interpenetrating double-network hydrogel and showed scanning electron microscopy images at different magnifications that revealed a multiscale porous structure. Figure 2d outlined the Bayesian optimization workflow that iteratively proposed formulations with expected improvement, maximum probability of improvement, and lower confidence-bound strategies, fabricated and tested new samples, and updated the dataset to minimize a composite property objective.

Supervised learning mapped formulation and process descriptors to properties that included swelling ratio, elastic modulus, toughness, adhesion energy, degradation rate, ionic conductivity, and release kinetics. Interpretable models on curated hydrogel sets predicted printable versus non-printable outcomes with balanced accuracy near 0.8 to 0.9 and recovered threshold rules in storage modulus and yield stress that aligned with filament collapse tests over 2 to 10 mm spans [38,60]. For tensile behavior, an interpretable gradient-boosted model trained on 350 hydrogel records reached coefficient of determination equal to 0.80 on held out data while ranking crosslink density and solvent fraction as leading factors [61]. In lithography datasets of 10^2^ to 10^3^ measured voxels, hybrid optical physics plus learning achieved root mean square error near 10 to 30 µm for cure depth and 5 to 15 percent for modulus [62,63].

Inverse pipelines posed composition and process selection to meet target rheology or adhesion subject to feasibility constraints. Surrogate-assisted Bayesian optimization reduced the number of experiments by roughly fifty percent compared to grid search on multi objective problems and identified Pareto improvements within 10 to 30 iterations on spaces with 10^3^ to 10^5^ candidates [64]. Reviews and case studies in extrusion reported that optimization of polymer mass fraction, temperature, and speed reduced pore size error from about 80 µm to about 30 µm within 20 to 25 trials while satisfying viscosity caps near 10^3^ Pa·s to protect cells [65]. Active learning surveys in materials science formalized acquisition policies and stopping rules that were directly transferable to hydrogel formulation loops [66]. Bayesian optimization and active learning enabled sample efficient exploration of composition–process–property trade spaces for hydrogel.

Rheology-informed classification and regression predicted strand width, interlayer adhesion, and defect likelihood for direct ink writing, while computer vision identified anomalies and guided parameter adjustment. Interpretable learning connected storage modulus in Pa, yield stress in Pa, and loss tangent to strand retention with F1 scores near 0.9 and distilled actionable ranges such as minimum yield stress near 50 to 100 Pa for 0.25 mm strands at 10 mm·s^−1^ [38,60]. In layer-wise monitoring during extrusion, convolutional models detected under-extrusion and over-extrusion with accuracy near 0.9 and supported adjustments that reduced strand width variance by about thirty percent [67]. In robotic gel printing, vision-based feedback improved placement precision and stability across tens of minutes of operation [43]. For inkjet processes, models built around the Ohnesorge and Reynolds groupings predicted stable droplet formation within Z equal to 1 divided by Oh near 1 to 10 at droplet diameters near 20 to 50 µm and velocities near 5 to 10 m·s^−1^ [45,68,69]. Integrating rheology-aware predictors with vision and control tightened process windows and yielded measurable quality gains in real time.

Machine learning also supported sensing and actuation with hydrogel in soft robotics and wound monitoring. Hydrogel-based strain and ionic sensors’ signals fed learning models for state estimation, with reported classification or regression accuracy above 0.9 for gesture recognition or pressure estimation over 10 to 100 kPa ranges [70,71]. Intelligent wound dressings used reactive oxygen species responsive gel matrices with machine learning analysis to track inflammation in real time and maintained cell viability while operating within safe optical or electrical exposure limits [72]. Hydrogel-based actuators and soft robots benefited from data-driven models that mapped control inputs to deformation with millimeter-scale positional error on centimeter-scale bodies [73,74]. In a cross-disciplinary example, a machine learning-enhanced soft robotic system inspired by rectal functions was developed to investigate fecal incontinence, illustrating how hydrogel-based sensing and actuation could be combined with data-driven control for physiologically relevant gastrointestinal function studies [75]. Responsive hydrogel systems paired with machine learning delivered adaptive sensing and control performance relevant to healthcare and soft machines.

Representations and features tailored to hydrogel were reviewed as follows. Formula-level features included extended connectivity fingerprints from simplified molecular input line entry system strings, counts of functional groups, degree of substitution in percent, mass fraction solids in percent, initiator type and loading in percent, solvent and ionic strength in mol·L^−1^, cure temperature in K, and cure route. Network and structure features included crosslink density inferred from rubber elasticity, mesh size estimated from Flory–Rehner, porosity from image analysis, and defect fraction from segmentation. Topology-aware descriptors from persistent homology quantified porous networks and correlated with transport. Process descriptors included mixing energy, ultraviolet dose in J·m^−2^, and printer parameters such as nozzle diameter in m, speed in m·s^−1^, and pressure in Pa. Optical features for vat printing used the Jacobs relation C equal to D_p_ times ln(E over Ec), where D_p_ and Ec were learned or measured, with typical D_p_ near 50 to 200 µm and Ec near 100 to 1000 J·m^−2^ for gelatin methacrylate and poly(ethylene glycol) diacrylate systems [76]. Inkjet features used Ohnesorge number Oh equal to µ divided by square root of ρσL and Z equal to 1 divided by Oh to encode jetting stability [45]. Physics-informed descriptors anchored models to transferable variables that generalized across chemistries and platforms.

The models’ classes applied in hydrogel were summarized. Tabular problems with 10^2^ to 10^3^ rows and tens of features favored tree ensembles and gradient boosting, where post hoc interpretation such as Shapley additive explanations revealed that a small set of rheological and optical variables dominated predictions [61]. Image-based links between microstructure and properties benefited from convolutional networks that reached defect detection accuracy near 0.9 at frame times below 0.1 s for extrusion monitoring [67]. Sequence or language models learned polymer representation directly from text like simplified molecular input line entry system and achieved multi-property prediction across millions of hypothetical polymers [6]. Physics-informed neural networks and multiphysics-informed models captured swelling and diffusion fields and reduced data needs by encoding conservation laws and constitutive relations [77]. Polymer Genome and related frameworks offered application-ready baselines with mean absolute errors competitive with experiments for several classes of polymer properties [78]. Ensemble learners excelled on small to medium datasets, convolutional networks handled structure images and monitoring, and physics-informed architectures extended validity beyond training data.

### 2.2. Artificial Intelligence Design for 3D Printability

Machine learning addressed the central difficulty that three-dimensional printability depended on a coupled set of formulation and process variables that spanned polymer chemistry, crosslinking route, rheology, and toolpath parameters [79,80,81,82,83,84,85,86]. Quantitative surveys showed that printable direct ink writing hydrogel typically used zero shear viscosity in the range 10^2^ to 10^4^ Pa·s and yield stress in the range 10 to 10^3^ Pa with nozzle diameters near 0.1 to 0.4 mm and speeds near 5 to 50 mm·s^−1^, while lithography-based systems introduced optical constraints defined by the working curve where cure depth C followed C equal to D_p_ ln(E/Ec) with penetration depth D_p_ and critical radiant exposure Ec as formulation-dependent parameters [38]. Reports on light-based networks documented D_p_ in the range 50 to 200 µm and Ec in the range 100 to 1000 J·m^−2^ for common hydrogel precursors, and highlighted site to site variability that motivated standardized, data-driven control [38,87]. The dimensionality and variability of the printability space favored data-efficient modeling that compressed exploration before experimentation.

**Figure 3:**
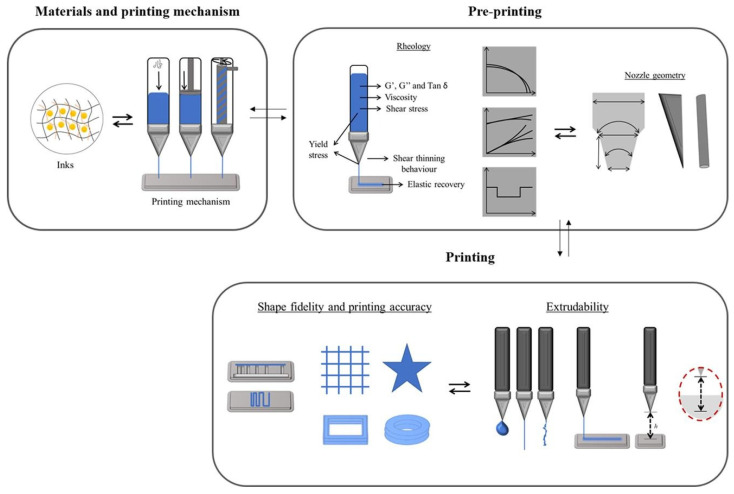
Interdependent material properties and process parameters in gel-based additive manufacturing, adapted with permission from ref. [88], Elsevier, CC BY-NC-ND 4.0.

AI optimized printability in hydrogel additive manufacturing by learning the links between gel rheology and process settings to ensure filament continuity, shape fidelity, and structural integrity, while tuning nozzle size, pressure, speed, and path height to the material’s viscosity, shear thinning, viscoelasticity and yield stress [88], as shown in Figure 3. It predicted and even inversely designed formulations that met target printability metrics using rheology-based inputs such as viscosity, G′ and G″, and it classified formulations as printable or non-printable to guide composition and parameter choices. In operation, AI further enabled adaptive, real-time monitoring and correction to prevent defects and maintain consistent layer quality in complex builds.

Supervised prediction for extrusion printability used physics-informed features derived from rheology and simple filament tests. Interpretable models that took storage modulus in Pa, loss tangent, yield stress in Pa, and recovery time in s as inputs separated printable from non-printable conditions with balanced accuracy near 0.80 to 0.92 on datasets of 10^2^ to 10^3^ labeled prints, and reproduced threshold behavior such as minimum yield stress near 50 to 100 Pa for 0.25 mm strands at 10 mm·s^−1^ to prevent mid-span collapse over 2 to 10 mm [3]. These reports used finite datasets with specific ratios of printable to non-printable cases and were usually carried out under a single laboratory protocol, so the quoted balanced accuracies reflected performance under controlled and partly imbalanced conditions rather than guaranteed cross-laboratory behavior. Later analyses of data readiness showed that changes in measurement protocol, imaging thresholds and unit harmonization could shift error by 10 to 30 percent when models were transferred between sites [3,38]. Studies that combined hierarchical features with process variables such as pressure in Pa, temperature in K, and speed in m·s^−1^ reported mean absolute error near 20 to 50 µm on 200 to 400 µm strand width targets and reduced false positives by incorporating recovery within 1 to 10 s after shear cessation [89]. Supervised learning achieved micrometer-scale error for geometric outcomes when models encoded rheology and basic process descriptors.

Inkjet printability benefited from dimensionless groups that summarized stability windows and thus created compact input spaces for learning. Analyses in droplet mechanics emphasized the Ohnesorge number Oh equal to µ divided by √(ρσL) and the printability parameter Z equal to 1 divided by Oh, which mapped viscosity µ in Pa·s, density ρ in kg·m^−3^, surface tension σ in N·m^−1^, and characteristic length L in m to stable jetting regimes. Experiments and reviews reported robust ejection for Z in the range 1 to 10 with droplet diameters near 20 to 50 µm and velocities near 5 to 10 m·s^−1^, while supply pressure tuning shifted velocity by only a few percent but improved stability and duty cycle [68,90]. Machine learning classifiers trained on experimental Oh, Reynolds, and Weber groupings separated satellite-prone regimes from stable ejection with accuracy near 0.85 to 0.95 on datasets containing 10^3^ shots per condition [91,92]. Embedding Ohnesorge and related groups produced compact models that predicted stable jetting across formulations and printers.

Vat photopolymerization printability relied on optical kinetics and dose control, which lent themselves to hybrid theory plus learning. The Jacobs working curve predicted C from exposure E and parameters D_p_ and Ec. Experiments with hydrogel resins showed that adding absorbers or varying initiator fraction shifted Ec by factors of two to five and enabled features near 50 to 100 µm at layer thickness near 25 to 50 µm with exposures in the range 100 to 500 J·m^−2^ [45,76]. Data-driven surrogates that took E, D_p_, Ec, and scattering indices from micrographs as inputs achieved root mean square error near 10 to 30 µm for cure depth and near 5 to 15% for elastic modulus on datasets with 10^2^ to 10^3^ measured voxels [45]. Coupling the working curve with learned corrections yielded sub 100 µm predictability for feature formation under varied formulations.

Process monitoring and control closed the loop between prediction and actuation. Layer-by-layer imaging with convolutional models detected under-extrusion, over-extrusion, and misalignment with accuracy near 0.90 at frame times below 0.1 s, and feedback reduced strand width variance by about 30 percent relative to open-loop control while holding a 200 to 300 µm set point [67]. Optical coherence approaches integrated with extruders measured filament size, layer thickness, and interlayer fidelity in situ, enabling automatic parameter nudging and repair of discontinuities during prints that lasted 10 to 30 min [93,94]. Studies of real-time inspection in laser-based printing suggested similar benefits for layer uniformity and depth control once dose and scan parameters were included as features [95]. Vision and optical coherence-based feedback paired with inference stabilized geometry and improved yield in real time.

Closed-loop design using Bayesian optimization and active learning accelerated convergence to printable windows and improved multi objective tradeoffs. Constrained multi objective policies identified Pareto improvements within 10 to 30 iterations on candidate spaces with 10^3^ to 10^5^ compositions and settings, and reduced experimental counts by roughly 50% relative to grid or factorial strategies [96,97]. In extrusion, expected improvement-based loops tuned polymer mass fraction, speed, and temperature to reduce pore size error from about 80 µm to about 30 µm within 20 to 25 trials while respecting viscosity caps near 10^3^ Pa·s to preserve cell outcomes. In inkjet, acquisition rules navigated Z in the range 1 to 10 at fixed nozzle sizes to maximize line continuity while penalizing satellites [96,97,98]. Learning formulations under uncertainty with Pareto-aware acquisition further balanced fidelity, modulus, and throughput without manual weight tuning [99]. Sample efficient active design reached practical geometric targets in tens of experiments while honoring feasibility constraints.

Data readiness and representation determined how well printability models generalized across chemistries and devices. Reports that harmonized units, temperatures, and illumination intensities and that recorded rheology methods and imaging thresholds reduced site effects that otherwise increased error by 10 to 30 percent when models trained in one laboratory were tested in another [3,38]. Polymer informatics resources that supported graph or fingerprint descriptors for monomers and crosslinkers, together with process features such as nozzle diameter in m, pressure in Pa, speed in m·s^−1^, and ultraviolet dose in J·m^−2^, enabled interoperable tabular datasets. Baseline predictors for polymer properties on such platforms delivered near-instantaneous inference and provided transfer starting points for hydrogel systems [50,78,100]. Careful curation and physics-aware feature sets improved cross-device performance and reduced retraining burden.

Theory guided features improved interpretability and robustness for three-dimensional printability prediction. Flow curves fit to Herschel–Bulkley or Carreau–Yasuda relations yielded yield stress in Pa, consistency index in Pa·s^n^, and relaxation time in s as inputs, while collapse tests over spans of 2 to 10 mm and corner sharpness metrics supplied labels for shape fidelity. Models that included these variables produced F1 scores near 0.9 for strand retention and mean absolute error near 20 to 50 µm for width prediction [89]. In vat systems, the Jacobian relation C equal to D_p_ ln(E/Ec) explained dose response, and machine learning residuals corrected for scattering and diffusion-driven departures from the logarithmic law. In inkjet, regimes organized by Oh and Z framed stability classification and regression for droplet velocity in m·s^−1^ and volume in nL [76]. Theory-anchored descriptors constrained models to physically valid regimes and improved extrapolation beyond the training set.

## 3. Recent AI Application in Hydrogel Additive Manufacturing

### 3.1. Reasons to Use Machine Learning

Machine learning addressed the combinatorial design space of hydrogel formulations and print settings that had previously required slow trial and error. Formulations mixed monomers, crosslinkers, solvents, salts, nanoparticles, and biological components in continuous ranges, while extrusion or light-based printing exposed them to interdependent process parameters. Interpretable models and graph-based representations learned from past experiments to expose non-obvious relations between chemistry, rheology, and print fidelity. Reported datasets already reached 150 to 180 unique hydrogel cases and identified 13 rheology features that governed print success, which motivated data-driven discovery over manual heuristics [57,68]. Machine learning reduced empirical screening by learning structure–process–property relations from hundreds of points rather than thousands, enabling more efficient exploration of hydrogel design spaces.

Machine learning enabled accurate prediction of intrinsic hydrogel properties that controlled performance and safety. Artificial neural networks classified temperature-responsive swelling states of poly N isopropylacrylamide hydrogel from synthesis parameters with a relative prediction error of 0.11 and supported targeted synthesis thereafter [101]. Graph neural network and graph convolutional approaches predicted polymer glass transition temperature with root mean square errors on the order of tens of kelvin and achieved approximately 5 K lower error than fingerprint-based neural networks for melting temperature, while also learning density in kg·m^−3^ and modulus in GPa from repeat unit graphs [102,103]. Deep learning predicted ionic conductivity of polymer electrolytes across temperature with high fidelity, which guided gel polymer electrolyte design for electrochemical devices [104]. A convolutional pipeline predicted elastic moduli directly from scanning electron microscopy images of hydrogel microstructure, linking mesoscale morphology to stiffness in kPa to MPa [44]. Because SEM images of lyophilized hydrogels were often affected by preparation artifacts, variable porosity, and imaging noise, this convolutional approach still depended on careful data cleaning, normalization, and inspection of saliency maps to avoid learning spurious texture cues. Without such preprocessing and curation, CNN filters could misinterpret noise or lyophilization-induced pores as mechanically relevant structure, which reduced generalization to hydrogels with different surface charge, contrast, or porosity. Predictive models delivered quantitative accuracy for swelling state, thermal transitions, density, stiffness, and ionic transport within errors small enough to guide formulation choices before synthesis.

Machine learning revealed general principles that linked bulk rheology to printability across diverse inks. An interpretable workflow trained on 180 formulations uncovered a minimal set of 13 rheology descriptors that predicted filament formation and shape fidelity across many chemistries [38]. A follow-up study compiled a rheology–printability database of 150 printed hydrogels and quantified the correlation between flow indices and successful strands, enabling direct screening by rheology alone [89]. High-throughput characterization integrated automated well plate rheometry with physics-guided learning to map viscosity–yield stress landscapes for gels at scale, accelerating data collection from many samples in hours rather than weeks [105]. Rheology-informed neural networks and related scientific machine learning respected constitutive equations while learning parameters, which improved generalization for complex fluids [106]. Rheology-informed machine learning replaced ad hoc print trials with predictive maps from shear history to filament stability, reducing failed prints and material waste.

Machine learning accelerated multi objective process optimization for hydrogel printing under competing goals of fidelity, throughput, and biological viability. Gaussian process models within surrogate-assisted Bayesian optimization located Pareto optimal regions of extrusion flow properties and print settings with limited experiments by balancing exploration and exploitation [64]. Parameter studies guided by support vector regression and population-based search identified optimal nozzle diameter, layer height, and coordinated speeds for sodium alginate gels at d = 0.6 mm, h = 0.3 mm, and 8 mm·s^−1^ for both print and extrusion speeds, producing continuous filaments and controlling die swell [43]. Broader reviews of bioprinting confirmed that Bayesian optimization and related probabilistic models consistently improved yield with modest data, which aligned with quality by design principles used in regulated manufacturing [65,107]. At the same time, GP-based Bayesian optimization frameworks became less reliable when the design space contained many coupled compositional and process variables: the number of experiments required to fit an accurate global surrogate grew rapidly with dimensionality, acquisition functions became harder to optimize, and the search could concentrate prematurely in a narrow region of the space. To remain sample efficient in such high dimensional composition spaces, recent surrogate-assisted multi objective optimization for rheological design decomposed the design space into lower dimensional subproblems and adopted localized Gaussian processes, trust-region Bayesian optimization, or ensemble Bayesian optimization variants [64]. Probabilistic optimization strategies achieved high-quality prints with tens of trials by targeting Pareto fronts over discrete grids of pressure, speed, and temperature.

Machine learning improved closed-loop quality control for gels during printing by combining sensing and control with inference. A model estimated ink rheology in situ from a simple printed test pattern, converting camera data to viscosity and yield stress estimates without interrupting production [108]. Computer vision detected deviations between commanded and deposited paths and then compensated robot motion, which increased filament path fidelity and reduced geometric error relative to nominal toolpaths [43]. Reviews of extrusion printing documented that monitoring-based learning pipelines adjusted pressure and speed in real time to sustain uniform strand width and interfilament spacing, protecting cell viability and shape fidelity [60,109]. In situ inference and vision-based feedback drove the process toward consistent bead geometry and uniform porosity without repeated offline calibration.

Machine learning supported inverse design and targeted functionality by linking chemistry and processing to application-level metrics. For bioelectronics and sensors, data-driven workflows connected monomer choice, dopant level, solvent fraction, curing schedule, and print pattern to ionic conductivity, modulus, and adhesion that satisfied soft interface requirements [60]. In conductive and responsive hydrogel, reported property targets included breaking stress near 0.19 MPa and breaking strain near 300 percent, along with response times near 200 ms, which created quantitative objectives for model-based design [110]. Reviews in artificial intelligence enabled hydrogel design summarized inverse tools that suggested candidate chemistries for gelation and self-assembly from small training sets, for example, nucleoside derivative libraries of size 71 that produced accurate gelation predictions [111]. Inverse design reframed formulation work as optimization against numeric targets such as modulus in kPa to MPa, response time in ms, and ionic conductivity in S·m^−1^, enabling direct search for fit to function.

Machine learning also integrated multiscale evidence, from molecular graphs to imaging and process trajectories, to deliver more transferable predictions. Multitask graph neural networks learned polymer property vectors across several endpoints at once and shared information across tasks, which reduced error and data requirements per property [112]. Recent work introduced unified multimodal representations that joined sequence, composition, processing, and microstructure, improving accuracy on sparse tasks that resemble hydrogel development [113]. Scientific machine learning with embedded constitutive constraints preserved physical structure and improved extrapolation when datasets were small, which matched typical hydrogel campaigns where data sizes ranged from tens to a few hundred points [105,106]. Cross-task and multimodal learning increased data efficiency and robustness for hydrogel problems where comprehensive datasets were not yet available.

Finally, machine learning linked material design with printability and cell response for biomedical objectives. Frameworks that combined rheological modeling, computational fluid dynamics, and regression predicted as extruded cell viability from wall shear stress magnitude and exposure time, allowing tuning of pressure and speed to maintain viability while preserving shape fidelity [60]. Systematic surveys across polymer and hydrogel bioprinting reported quantitative classification and regression of printability with reported coefficients of determination up to approximately 0.78 for certain models, and highlighted that neural network-based approaches captured nonlinearity between formulation and outcome [112]. Cell-focused high-throughput printing platforms enhanced with learning tuned five or more interdependent parameters to achieve uniform droplets and consistent cell numbers per voxel, which raised reproducibility for tissue models [109]. Data-driven models enabled co-optimization of filament quality and biological viability by predicting stresses and survival, supporting safer and more reproducible constructs.

Machine learning provided quantitative, theory-informed tools that predicted hydrogel properties, mapped rheology to printability, optimized process parameters with few experiments, enabled closed-loop control, and supported inverse design against numeric performance targets. These capabilities reduced development cycles from weeks to days, cut print failures, and improved reproducibility for hydrogel additive manufacturing [110,111,114]. However, there are also few failure cases and limitations of machine learning. Convolutional pipelines that inferred elastic modulus directly from scanning electron microscopy images of lyophilized hydrogels sometimes struggled when the input images lay outside the training distribution, for example, when lyophilization artifacts distorted pore size and connectivity or when non-porous hydrogels were presented, leading to inaccurate predictions and underscoring the need for careful preprocessing and dataset curation [44]. Surrogate-assisted Bayesian optimization frameworks based on Gaussian processes became more difficult to scale as the number of tuned formulation and process variables increased, motivating strategies that decomposed high-dimensional design spaces into lower dimensional subproblems and employed localized or trust-region Gaussian processes instead of expensive global surrogates [64]. Broader analyses of extrusion bioprinting and materials design further reported that tree-based and other supervised models could lose predictive accuracy when model knowledge was not transferable to new datasets and could exhibit asymmetric performance when predicting multiple responses, highlighting that models trained on small or biased datasets may give overconfident yet inaccurate predictions away from the training domain [25,26]. Reviews in polymer and hydrogel informatics therefore recommended calibrated uncertainty quantification, ensemble baselines, and physics-guided priors to flag out-of-distribution conditions and to delineate regimes where machine learning recommendations should be treated with caution [53].

### 3.2. Wide Availability of Resources

Public data platforms for polymers supplied large, traceable corpora that supported hydrogel informatics. A curated dataset of 1073 polymers with density functional theory-derived structures and properties expanded access to band gap in eV and dielectric constant values and was openly downloadable with 4292 calculation records, which enabled transfer of methods to hydrogel design tasks [115]. Polymer Genome offered instant machine learning predictions for property sets after community curation of training data and documentation, which lowered barriers for non-specialists to screen candidate hydrogel building blocks [78]. A benchmark called PI1M aggregated on the order of one million polymer entries for model evaluation and provided a route to reproducible train test partitions for hydrogel-relevant endpoints [116]. The PoLyInfo program reported more than 0.5 million polymer data points with processing metadata and measurement conditions that were vital for reproducible gel property comparisons [46,117]. Widely accessible polymer informatics platforms and datasets provided thousands to millions of entries that accelerated hydrogel formulation screening and model benchmarking with reproducible splits.

The community also gained specialized descriptor and simulation resources that closed gaps for copolymers and amorphous networks. A copolymer descriptor database compiled 24 descriptors across reactivity, electronic, geometric, and conventional categories for 2500 radical monomer pairs from 50 monomers, which supported predictive models for composition to property relations relevant to gels [118]. The RadonPy framework automated all atom molecular dynamics workflows to compute fifteen properties for more than 1000 amorphous polymers in parallel, which supplied density in kg·m^−3^, refractive index, thermal conductivity in W·m^−1^·K^−1^, and other targets that informed gel component selection [49]. Descriptor libraries and automated simulation pipelines provided structured inputs and high-volume synthetic labels that improved data coverage for hydrogel modeling.

Open hardware and low-cost fabrication workflows made hydrogel printing methods broadly available. A freely documented hybrid bioprinter enabled alternating extrusion of hydrogel with fused filament deposition and allowed multi-material constructs using commodity components, which reduced entry cost while preserving toolpath control [119]. A microfabrication pipeline combined consumer vat photopolymerization with polydimethylsiloxane soft lithography and produced channel features from 20 µm to 50 µm and centimeter-scale structures for tissue culture devices, demonstrating that precise molds and masters could be produced without specialized facilities [120]. A survey of hydrogel printing practices summarized common desktop printer capabilities and typical parameter windows, which supported consistent transfer of process recipes between laboratories [88]. Open hardware and desktop workflows expanded practical access to multi-material hydrogel printing and microfabrication with feature sizes down to 20 µm.

Community guidelines and quantitative printability protocols standardized measurements that could be shared across groups. A comprehensive review synthesized shape fidelity metrics and practical tests for extrusion and light-based printing and connected them to rheological quantities such as yield stress and storage modulus, enabling laboratories to compare hydrogel inks without bespoke fixtures [3]. A quantitative study of pentanoate-functionalized hyaluronic acid used three recommended rheological protocols to define printability thresholds and provided repeatable criteria for gel formulation choices [121]. An essential rheology guide reported viscosity values from 10^−3^ Pa·s to 6 × 10^4^ Pa·s across extrudable gels and related these to shear thinning and viscoelastic responses that governed filament formation [4]. A rheology to manufacturing perspective clarified how storage modulus G′ and loss tangent mapped to strand stability and part integrity in three-dimensional printing [122]. Shared rheology protocols and printability metrics enabled laboratories to publish comparable hydrogel measurements across six orders of magnitude in viscosity and well-defined moduli.

Design and optimization toolkits that had proven value in polymer science became directly usable for gel processes with small experiment counts. A multi objective Bayesian optimization system guided flow copolymerization and identified Pareto optimal tradeoffs for targeted performance, demonstrating efficient search under limited trials that mirrored hydrogel printing campaigns [123]. A data-efficient terpolymer study executed two optimization iterations across 89 synthesized terpolymers and balanced glass transition temperature against monomer incorporation, illustrating practical experimental budgets for exploratory design [124]. A machine learning study of multicomponent supramolecular materials used Bayesian optimization to target noncovalent assemblies, which provided a template for hydrogel network tuning with limited data [125]. A review of extrusion of soft materials summarized quantitative relations between pressure-driven shear stress and cell survival, reporting cases with 90% viability near 40 Pa and significant loss near 250 Pa and thereby enabling constraint-based design of print settings [126]. Optimization frameworks and quantitative constraints enabled targeted hydrogel design in tens to low hundreds of experiments while maintaining viability limits in the range of 10^1^ to 10^2^ Pa.

Process physiology and viability maps became widely available through open access reviews and mechanistic modeling that translated to practice. A synthesis of bioprinting shear and hydrostatic effects explained how nozzle geometry, flow rate, and ink rheology set wall shear stress and residence time and reported guidance for mitigating damage during printing [127]. A lattice Boltzmann and finite element study predicted deformation of cells inside and at the exit of nozzles with explicit stress fields and offered quantitative stress trajectories for process design [41]. A rheology-focused guide for extrusion reported minimal effect on viability below approximately 5 kPa with viability up to 96% and recommended monitoring shear rate histories to avoid damage at higher stresses [4]. Openly available modeling and review resources delivered quantitative safe operating envelopes for hydrogel bioprinting, such as viability near 96% below 5 kPa.

Representational standards improved data interoperability and curation for gels and polymers that feed hydrogel design. BigSMILES introduced a structural line notation that represented stochastic macromolecules and enabled consistent capture of repeat unit distributions and branching in machine-readable strings, which supported fair data principles for hydrogel components [47]. PoLyInfo articles documented machine-readable schemas for polymer knowledge and emphasized consistent capture of processing and test conditions, which reduced ambiguity when merging hydrogel property reports across sources [46]. Standard representations and schemas reduced data loss during sharing and supported automated parsing of polymer structures and metadata used in hydrogel.

Resource reviews of hydrogel classes and printing approaches aggregated material chemistries and performance ranges that supported rapid shortlisting. A recent overview summarized natural and synthetic hydrogel families for tissue engineering and reported tradeoffs in mechanics and degradation, which facilitated selection of bioink backbones and blends [128]. Work on smart alginate inks identified an optimal effective kinematic viscosity window near 400 to 3000 mm^2^·s^−1^ for stable deposition of a tuned system, which translated to ranges in Pa·s via density for practical nozzle sizing [129]. An earlier broad survey cataloged characterization protocols and process settings for hydrogel three-dimensional printing and connected rheology to filament stability, which aided replication of results between labs. Consolidated reviews and ink-specific studies provided numerical windows for viscosity and moduli that shortened the path to printable hydrogel recipes.

Foundational theories furnished transferable models that underpinned data interpretation and design rules across resources. Flory and Rehner described swelling of crosslinked networks by balancing elastic free energy with mixing free energy, which still guided extraction of crosslink density from equilibrium swelling ratios and informed comparisons between gel datasets [130]. The Herschel–Bulkley constitutive relation characterized yield stress fluids with $\tau =\tau_{0}+K{\dot{\gamma }}^{n}\;$ and was widely used to map pressure and flow rate to shear rate in nozzles, enabling translation of printer settings to expected stresses in Pa for diverse inks. The combination of network swelling theory and yield stress rheology supplied simple equations that converted raw measurements and printer settings into comparable parameters across studies and repositories.

Overall conclusion for this section, openly available datasets, descriptor libraries, simulation pipelines, hardware designs, quantitative protocols, optimization frameworks, viability maps, representation standards, and transferable theories created a mature resource ecosystem for hydrogel materials and hydrogel additive manufacturing. These resources reported feature sizes as small as 20 µm for molds, viscosity ranges from 10^−3^ Pa·s to 6 × 10^4^ Pa·s for printable inks, viability near 96% below 5 kPa, and dataset sizes from 1073 entries to more than 0.5 million records, which together enabled reproducible and efficient discovery and manufacturing. Table 2 concisely maps open-source packages to employed models, dataset sizes and types, hydrogel application focuses, reported quantitative metrics, and references.

### 3.3. AI Applications in Hydrogel Additive Manufacturing

Artificial intelligence linked rheology to direct ink writing outcomes and replaced manual print trials with predictive screening. Interpretable learning on 180 formulations identified thirteen rheological descriptors that governed strand formation across chemistries and provided quantitative thresholds for yield stress and storage modulus that aligned with extrusion theory based on the Herschel–Bulkley law $\tau =\tau_{0}+K{\dot{\gamma }}^{n}$ [38]. A complementary study assembled a rheology–printability database of 150 printed hydrogels and reported that machine learning models separated horizontal and vertical printability regimes that reflected post-deposition recovery versus high strain rate flow in the nozzle [39]. Review work on ink rheology confirmed that printable systems typically exhibited shear thinning with viscosity spanning 10^−3^ to 10^4^ Pa·s, which set feasible pressure and speed windows for extrusion [136]. Data-driven models trained on 150 to 180 cases captured process–structure rules from standard rheology and enabled rational selection of print settings before fabrication.

**Figure 4:**
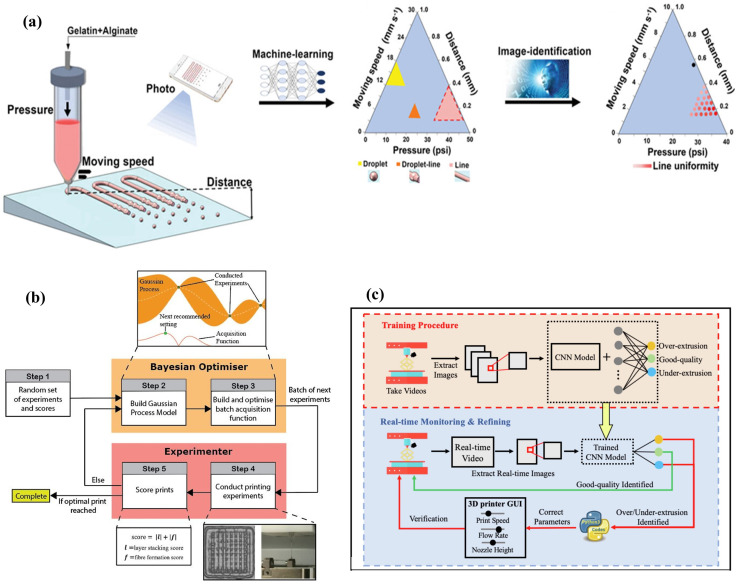
AI application in hydrogel additive manufacturing. (**a**) The AI high-throughput system screens the parameters for bioprinting through the phase diagram and optimizes the extrusion form and the uniformity of the lines [137]. (**b**) The Bayesian optimization framework is applied to the optimization of bioprinting [138]. (**c**) The CNN model predicts the monitoring, optimizes the classification, and automatically adjusts the printing parameters accordingly [139]. Panel (**a**) adapted with permission from ref. [137], Wiley, CC BY 4.0. Panel (**b**) adapted with permission from ref. [138], Elsevier. Panel (**c**) adapted with permission from ref. [139], Elsevier.

Figure 4a outlined an AI-assisted high-throughput printing-condition screening system (AI-HTPCSS) [137] that couples a programmable pneumatic extrusion (bio)printer with an image-analysis algorithm to rapidly map phase diagrams of pattern states and line uniformity across printing pressure, speed, and distance. It then uses the optimized conditions to print uniform multi-layer grid hydrogel scaffolds, which are applied as dressings to accelerate diabetic wound healing. Figure 4b showed a closed-loop workflow in which a Bayesian optimizer proposed batches of new printing settings after an initial random set of scored experiments. The optimizer built a Gaussian process model, optimized an acquisition function shown in the inset, and recommended the next settings for the experimenter. The experimenter conducted prints, scored them using layer stacking and fiber formation to form a composite score, and repeated the loop until an optimal print was reached [138]. Figure 4c presented the workflow of an autonomous FDM monitoring and correction system with a training stage, real-time monitoring, and a refining loop [139]. During operation, real-time images were fed to a saved CNN that classified under-extrusion, good quality, and over-extrusion, and the system adjusted printing parameters automatically when defects were detected.

Artificial intelligence supported process optimization with modest experiment counts using surrogate modeling and Bayesian optimization. A surrogate-assisted multi objective optimization framework for extrusion targeted competing goals of fidelity and throughput and demonstrated efficient search across pressure, speed, and temperature with a small number of prints, formalizing the explore–exploit tradeoff [64]. In a related study, neural network models combined with Bayesian optimization predicted and improved cell survival while tuning nozzle diameter, pressure, and speed for gelatin and alginate systems and achieved coefficient of determination 0.71 for regression and 0.86 for classification on viability [40]. Optimization studies for polymer additive manufacturing further showed that Bayesian strategies located parameter sets that increased resolution and mechanical performance with few evaluations, providing templates for hydrogel campaigns [140].

Computer vision and learning closed the loop between sensing and control to improve geometric accuracy during printing. A vision-based compensation scheme adjusted robotic toolpaths from layer images and reduced the printing layer width disparity to 0.15 mm from 0.60 mm while lowering the average error area for curved filaments to 7.0 mm^2^ from 12.7 mm^2^ [43]. Vision-assisted extrusion control in additive manufacturing also quantified real-time filament width and extrusion rate, allowing pressure and speed adjustments that stabilized bead geometry [141]. Earlier work in additive manufacturing reported trajectory deviation detection and correction using learned image features, which translated directly to hydrogel strand placement [142]. Computer vision-guided feedback delivered measurable error reductions on the order of 40 to 75 percent in width and area metrics and provided practical recipes for adaptive path planning in soft materials printing.

Artificial intelligence models quantified the link between flow fields and cell response during extrusion and provided constraints for safe deposition. A study in extrusion bioprinting combined neural networks with Bayesian optimization to maximize viability and reported classification accuracy 0.86 while exploring multi-parameter settings [40]. A synthesis of hydrogel extrusion rheology found that cell viability remained high near 91 percent for stress levels around 5 to 10 kPa and dropped toward approximately 76 percent above 10 kPa, establishing numerical limits for controller design [4]. Lattice Boltzmann and finite element simulations resolved cell deformation inside and at the nozzle exit and highlighted the importance of extensional stresses that complemented shear estimates from the analytical solution for shear thinning fluids [41,143]. Machine learning and mechanistic flow models together defined operating envelopes by linking pressure and shear history to quantitative survival targets between 76 and 96%.

Artificial intelligence predicted hydrogel viscosity and other rheological properties from formulation variables and enabled inverse design of inks. Models trained on hybrid gelatin methacryloyl and hyaluronic acid data forecast viscosity across shear rates and guided composition toward print-ready windows with reported improvements against baseline regressors [144]. Data-driven viscosity predictors for multicomponent alginate–gelatin–cellulose systems used decision tree and random forest models across shear rates from 0.1 s^−1^ to 100 s^−1^ and provided explicit recipes that reached target viscosities [145]. Materials design reviews outlined inverse screening of gel formulations driven by graph-based polymer representations and experimental feedback, which tightened iteration cycles for soft materials [146]. Supervised learning on data sizes from tens to low hundreds delivered viscosity predictors across 0.1 to 100 s^−1^ that supported inverse formulation toward printable regimes.

Artificial intelligence connected process parameters to filament geometry and resolution with quantitative accuracy. A study in material extrusion predicted filament width and height from nozzle diameter, nondimensional nozzle height, pressure, and speed, and reported best test set coefficients of determination 0.90 for width and 0.96 for height over ranges of 0.47 mm to 1.79 mm and 0.20 mm to 0.66 mm [147]. In hydrogel bioprinting, hierarchical models informed by rheology improved prediction of printed feature resolution relative to concentration or parameter only baselines, demonstrating the benefit of physics-informed feature hierarchies [148]. Models achieved R^2^ near 0.90 to 0.96 for filament dimensions and confirmed that rheology-informed features enhanced generalization for hydrogel strands.

Artificial intelligence bridged microstructure and mechanics by learning from imaging data. A pipeline trained on scanning electron microscopy images predicted elastic modulus of hydrogel within the correct order of magnitude and extracted texture features indicative of pore size and connectivity, which provided a route to non-destructive stiffness estimation [44]. Finite element-augmented learning also predicted composite hydrogel properties from structural parameters, supporting simulation-informed data augmentation when experiments were scarce [149]. Image-based and simulation-assisted learning generated surrogate maps from microstructure to modulus and strength and reduced the need for destructive testing.

Artificial intelligence frameworks extended to four-dimensional and inkjet strategies that required time-dependent and droplet-scale control. Analyses of four-dimensional hydrogel printing emphasized model-based coupling of swelling kinetics with print architecture, where data-driven surrogates accelerated search through stimulus response spaces quantified in seconds to minutes [150]. Reviews of inkjet hydrogel printing documented artificial intelligence-assisted droplet formation control, linking waveform and viscosity to satellite droplet suppression and resolution in the 10 µm to 50 µm range [45]. Artificial intelligence accelerated design for time varying and droplet-based processes and helped achieve sub 50 µm features and targeted response times through surrogate evaluation of waveform and chemistry combinations.

Theory-guided models improved extrapolation and interpretability for hydrogel printing. The Herschel–Bulkley law converted pressure and nozzle geometry to shear rate histories inside cylindrical dies and explained measured thresholds such as critical yield stress 371 Pa and critical shear rate 100 s^−1^ for representative hydrogel [151]. The Carreau–Yasuda model provided a compact description of shear thinning across decades in τ and underpinned analytical solutions for flow in nozzles and the design of speed–pressure couplings used in controllers [143]. In this framework, the Herschel–Bulkley law $\tau =\tau_{0}+K{\dot{\gamma }}^{n}$ described how the shear stress τ increased with shear rate $\dot{\gamma }$, where the yield stress $\tau_{0}\;$ was the minimum stress needed for the gel to start flowing, the consistency index K  set the overall stress level, and the flow index n  controlled how strongly the viscosity decreased with shear rate (shear thinning). The same solid-like or liquid-like behavior could also be described by the linear viscoelastic quantities storage modulus G’, loss modulus G″, and their ratio, the loss tangent tanδ=G″/G’. The Carreau–Yasuda model introduced a characteristic time scale λ, which indicated how quickly the viscosity changed with shear rate and was consistent with the recovery times obtained from step-strain tests. Flory–Rehner-based network theories continued to support inverse mapping from swelling ratio to crosslink density, which informed targeted mechanical windows for printed constructs [152]. Embedding Herschel–Bulkley, Carreau–Yasuda, and Flory–Rehner relations into learning pipelines grounded predictions in physical parameters that could be transferred across printers, geometries, and gels.

Comprehensive syntheses in hydrogel additive manufacturing showed that artificial intelligence had matured from concept to practical guidance across materials, process, and quality. Reviews of hydrogel rheology emphasized quantitative links between viscosity, G′ to G″ ratio, and strand stability and provided stress–viability maps that practitioners used as constraints in optimization [122]. Overviews of direct ink writing showed that learning-based pipelines reduced empirical tuning and supported reproducible recipes across natural and synthetic systems by sharing parameter windows and error bars [153]. The field progressed toward data sharing and standardized metrics that enabled cross-lab model reuse and accelerated translation to complex constructs.

Overall conclusion for this section, artificial intelligence in hydrogel additive manufacturing delivered predictive printability from rheology, optimized parameters with small experiment counts, enabled vision-guided corrections with millimeter-level improvements, mapped flow histories to viability, predicted viscosity and strand geometry with R^2^ up to 0.96, inferred stiffness from images, and leveraged physical laws such as Herschel–Bulkley, Carreau–Yasuda, and Flory–Rehner to improve robustness. Together, these capabilities shortened development cycles and increased reproducibility across data sizes from tens to 180 cases and beyond.

Table 3 surveyed how artificial intelligence had been embedded across hydrogel additive manufacturing and highlighted method process pairings and typical targets. Supervised vision with convolutional neural networks enabled in situ defect detection for vat photopolymerization using DLP and SLA, while generative adversarial networks produced realistic synthetic data that enriched these imaging workflows. For extrusion-based DIW, Bayesian optimization accelerated tuning of temperature, pressure, and speed, and classical learners such as decision trees and random forests, often used with deep learning or polynomial fits, predicted printability as well as rheology and viscosity. In DLP and SLA, artificial neural networks and broader deep learning models predicted rheological properties and composition, and graph neural networks moved upstream to design inks from molecular structure and unlocked new material systems. Inkjet studies focused on process physics and used random forest, least absolute shrinkage and selection operator (LASSO), XGBoost, and support vector regression to predict droplet velocity and volume, and combined random forest, artificial neural networks, and support vector machines to forecast printability and drug dose. Together, these efforts showed a progression from defect monitoring to material and process co-design, with models selected to match data types that ranged from images and molecular graphs to scalar rheology and kinematic outputs.

## 4. Outlook

Artificial intelligence has already connected formulation space, rheology, and shape fidelity for hydrogel bioinks, and it has done so with quantitative targets that printing teams can verify.

**Figure 5:**
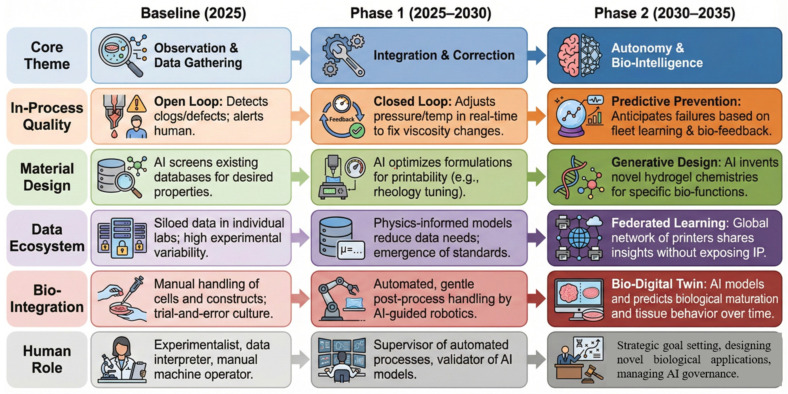
Strategic roadmap for artificial intelligence integration in hydrogel additive manufacturing (2025–2035).

Figure 5 provides a strategic outlook for the evolution of Artificial Intelligence in hydrogel additive manufacturing from a 2025 baseline through 2035. The roadmap illustrates a profound transition from the current state of isolated observation and manual data collection toward fully integrated, autonomous bio-intelligent ecosystems. Over the coming decade, the paradigm shifts from reactive, open-loop quality checks to real-time, closed-loop correction (Phase 1, 2025–2030), ultimately culminating in predictive fault prevention based on fleet learning (Phase 2, 2030–2035). Concurrently, material development advances from screening existing databases to generative design of novel chemistries, while data ecosystems mature from siloed laboratories into global federated learning networks. This technological trajectory significantly redefines the human element, elevating the operator’s role from manual experimentalist to a strategic supervisor governing autonomous processes and managing advanced concepts such as bio-digital twins.

Foundational studies on hydrogel printability mapped the influence of air pressure, feed rate, and nozzle distance on line width and lattice collapse, and showed workable windows near 37 °C nozzle temperature and −5 °C substrate temperature for alginate with gelatin support, which provided a reproducible baseline for data collection [162]. Light-based strategies increased speed and geometric complexity; for example, volumetric additive manufacturing of silk sericin and silk fibroin achieved complete objects in 60 to 165 s at 3 mW cm^−2^ with 2.5 to 10% protein content and Jaccard similarity indices near 0.7 to 0.84, while digital light processing refinements improved fidelity constraints for cell-compatible curing [163,164,165]. These process envelopes defined the measurable outputs that machine learning models could learn, such as line width error, filament sag distance, and overcure depth, and created space for inverse design. Quantitative printability envelopes provided the target variables that artificial intelligence needed to optimize hydrogel printing. Beyond these technical advances, future artificial intelligence-enabled hydrogel manufacturing will benefit from explicit ethical and reproducibility frameworks that follow FAIR data principles and promote model transparency. Under FAIR practices, researchers use persistent identifiers and rich metadata for formulations, rheological protocols, and print settings. They also provide open access to benchmark datasets, code, and trained weights whenever possible, together with standardized reporting of training, validation, and test splits, hyperparameters, and uncertainty estimates for predictive models. Clear documentation of training data provenance, model structure, and intended use improves the auditability and reproducibility of artificial intelligence tools, while ethical oversight and appropriate consent are particularly important when bioprinted constructs contain patient-derived cells or data. If such practices are embedded in hydrogel printing studies, they will support accountable deployment of machine learning models and smooth translation of automated workflows into regulated manufacturing and clinical environments.

Artificial intelligence enabled inverse design for composition and dose by coupling surrogate models with Bayesian optimization while respecting biological constraints. Volumetric silk printing demonstrated that light transport and Beer–Lambert predictions set penetration depth and voxel size, which the optimization loop treated as hard constraints to maintain feature size below the measured minimum cube edge while achieving printing times below 200 s [165]. High cell density strategies that used spheroids provided complementary constraints from biology, with a high throughput system printing approximately 600 chondrogenic spheroids into 1 cm^3^ constructs in less than 40 min and maintaining viability above 90%, which set objective terms for speed and viability in multi objective design [166]. Early aqueous hydrogel photoprinting and aspiration-assisted placement further showed how diffusion limited crosslinking and aspiration force thresholds shaped feasible regions for nozzle speed and pressure, which informed physics-informed priors in the optimization [167,168]. Multi objective surrogate models that encoded optics and transport delivered faster feasible searches while meeting ≥90% viability and sub-minute to minute-scale build times.

Closed-loop monitoring moved from post hoc inspection to process integrated sensing, which aligned well with artificial intelligence control. In situ ultrasound monitoring during extrusion of alginate with gelatin resolved layer bonding, transient elastic modulus, and surface roughness with depth resolution approaching 0.78 λ, and identified an optimal crosslinking time that avoided geometric distortion, which provided dense labels for learning controllers [169]. Optical assessment and modeling of extrusion bioprinting supplied complementary datasets for line width and defect states, and cellular automata captured post-printing cell behavior over 11 d in collagen-based constructs, which connected process signatures to biological outcomes [170,171]. Computer vision guidance for bioprinting in bone research outlined how accuracy and survival could improve with camera feedback, thus offering practical error classes for classifier training and thresholding in feedback loops [172]. In situ ultrasound and vision provided process-rich features that enabled artificial intelligence to correct flow, speed, and exposure during printing rather than after failure.

Artificial intelligence linked nozzle or voxel-scale choices to organ level function through cross-scale benchmarks that could be reproduced. Multicellular bioprinted skin grafts improved epithelialization and reduced scar metrics in small animals, and engineered edgeless skin constructs reported geometry-dependent mechanics, which together suggested modulus and anisotropy targets for printed hydrogel that artificial intelligence could pursue, for example, storage modulus windows in the 0.3 to 10 kPa range and dimensional drift below 5% after swelling [173,174]. Collagen-based internally perfusable scaffolds achieved high-resolution channels and perfusion, which connected print path and curing dose to mass transport targets such as perfusion capable channels below 200 µm and pressure drops within physiological limits [175]. Bacteria-laden hydrogel printing showed how spatial organization controlled function in living composites, motivating design variables such as local porosity between 20% and 60% and diffusion lengths below 100 µm to sustain activity [176]. Cross-scale targets connected artificial intelligence decisions to tissue-level performance metrics that could be verified experimentally.

Method development in photochemistry and path planning provided new levers for artificial intelligence to tune, with explicit numeric tradeoffs. Photoinhibition that combined simultaneous photoabsorption and radical control improved hydrogel photopatterning fidelity, reducing overcure depth and line broadening while maintaining cell activity, and provided dose-rate and inhibitor-concentration pairs that a model could predict within acceptable error bands [163]. Adaptive and context-aware volumetric printing reframed the slicer from layers to fields, shifting optimization from layer height and hatch spacing to light-field design and projection sequence, and creating a path to reduce support use while cutting build times by multiples for appropriate resin kinetics [164]. Tunable porosity analyses in light-based hydrogel printing highlighted viscosity modulators and resin composition as levers to achieve target pore sizes and branch diameters in vascular models, which could be encoded as constraints in artificial intelligence-supported slicing [177]. Advances in photochemistry and volumetric toolpaths made resin dose and porosity predictable control variables for artificial intelligence-guided design.

Looking forward, integration of foundation-scale polymer models with closed-loop bioprinting architectures could further streamline hydrogel design and deployment. Large curated resources and benchmarks for polymers, including BigSMILES-based representations, PoLyInfo, RadonPy, Polymer Genome, and PI1M, have already enabled graph- and fingerprint-based models that learn composition–property relations at scale and provide near instantaneous predictions for thermal, mechanical, and transport properties [46]. Coupling such pretrained polymer models to hydrogel printability surrogates would allow autonomous systems to propose candidate networks and formulations that satisfy rheological and photochemical constraints before experimental screening. On the control side, the closed-loop Bayesian and active learning schemes that now tune extrusion and inkjet parameters under fidelity and viability constraints [40] point toward physics-informed reinforcement learning controllers that embed constitutive laws and flow models directly into the policy, building on existing combinations of rheology, computational fluid dynamics, and scientific machine learning with embedded constraints [41]. In this setting, multi objective optimization would remain central, with Pareto-aware acquisition and reward formulations balancing geometric fidelity, throughput, and cell survival in real time during bioprinting [64]. As vision-based monitoring, optical coherence sensing, and high-throughput screening platforms continue to mature [67], these foundation polymer models, physics-informed reinforcement learning agents, and multi objective optimizers together could enable genuinely self-driving hydrogel bioprinting systems that close the loop from molecular design to process control and functional constructs.

Data-centric workflows emerged around bioprinted disease models and polymer informatics, enabling artificial intelligence to generalize across chemistries and printers with bounded error. Integration of three-dimensional bioprinting with multi-algorithm machine learning in glioma models reproduced patient molecular features and drug responses, which suggested that formulation and process descriptors could predict therapeutic readouts for complex tissues [178]. Applied machine learning for polymer discovery documented transferable embeddings for composition to property mapping and referenced bioink formulation tasks, providing routes to reduce prediction error on rheology or modulus to below ten percent with diverse training data [179]. Together with extrusion optical datasets and ultrasound signatures, these efforts identified printability cards that included viscosity versus shear rate, yield stress, filament sag distance, minimum channel diameter, cell survival, and imaging-derived defect probabilities for model training.

## Figures and Tables

**Table 1:** Additive manufacturing methods and process parameters.

AM Technology	Printing Types	Printing Parameters
Material extrusion	Direct ink writing (DIW) [7]	Shear thinning behavior, Viscoelastic modulus, Yield stress [8]; Nozzle diameter, Extrusion pressure, Printing speed [9], Extrusion pressure [10], Crosslinking [11]
Material jetting	Ink jetting [12]	Viscosity [13], Surface tension [14], Shear thinning behavior [15], Nozzle diameter [16], Cytocompatibility [17], Crosslinking [18], Driving waveform [19], Ejection frequency [20], Line stability [21]
Vat photopolymerization	Digital light processing (DLP) [22]	Photoinitiator type and concentration, Light wavelength [23], Light intensity, Exposure time [24], Pre-polymer type and concentration [25], Additives [26], Cell concentration and type [27], Post-printing processes [28], Layer thickness [29]
	Stereolithography (SLA) [30]	Photoinitiator type and concentration [31], Laser wavelength, Laser power, Scanning speed [32], Exposure energy [24], Layer thickness [33], Prepolymer concentration, Cell parameters [30]
Laser-assisted bioprinting	Laser-induced forward transfer (LIFT) [34]	Laser wavelength, Fluence [35], Donor film thickness, Viscosity, Surface tension [36], Donor-receiving substrate spacing, Scanning speed and repetition rate [37]

**Table 2:** Open-source packages, machine learning models, and dataset for hydrogel additive manufacturing.

Open-Source Package	Model	Dataset Size and Type	Application	Metrics	Ref.
Scikit learn, XGBoost, Optuna	Gaussian process, random forest	180 formulations, protein-inspired	Underwater adhesion optimization	Adhesion exceeded 1 MPa	[131]
Scikit learn	Interpretable classifier	180 formulations, rheology indices	Printability from rheology	Thirteen key rheology descriptors	[38]
Scikit learn	Multi-model classifiers	150 printed hydrogels	Rheology to printability mapping	Curated cross-chemistry mapping	[39]
Scikit learn	Ensemble classification	1568 bioprinting assays	Multi-property print quality	Sixteen properties standardized	[132]
Scikit learn	Neural network classifier	Literature PNIPAAm set	Swelling state prediction	Reported discrete state accuracy	[101]
PyTorch	Multiphysics-informed deep learning	Experimental swelling curves	pH-responsive swelling modeling	Reduced error versus baselines	[77]
Scikit learn	Hierarchical model	Suspended bioprinting set	Resolution prediction	Rheology-informed hierarchy improved	[133]
Scikit learn	Surrogate Bayesian optimization	Experimental prints, tens of trials	Process parameter optimization	Pareto improvement shown	[98]
Scikit learn	Vision-based regression	In situ test patterns	Rheology estimation from prints	Viscosity and yield stress predicted	[108]
Open-source hybrid bioprinter	Empirical mapping plus regression	Multiple gel prints reported	Dual material biofabrication	Open hardware enabled datasets	[119]
Scikit learn	Decision tree, neural network	Urea gelator library	Supramolecular gelation prediction	Physically motivated descriptors	[134]
Scikit learn	Quantitative structure models	Dipeptide gelator set	Gelation propensity classification	First predictive success reported	[135]
Scikit learn	Mixed regression models	Ten hydrogels, varied prints	Print width and porosity	Unified integrity metric used	[132]
Scikit learn	Random forest, SHAP analysis	180 formulations, rheology	General printability principles	Cross-chemistry transfer shown	[38]
Scikit learn	Regression on imaging features	Hydrogel SEM image set	Modulus prediction from images	Elastic moduli predicted	[132]

**Table 3:** The artificial intelligence methods and applications for hydrogel additive manufacturing.

AI Method Model	Applications	AM Methods	Ref.
Convolutional Neural Network (CNN)	In situ defect detection	Digital Light Processing (DLP), Stereolithography (SLA)	[154]
Generative Adversarial Networks (GAN)	Generate new samples that closely approximate the distribution of real data	Digital Light Processing (DLP), Stereolithography (SLA)	[155]
Bayesian Optimization (BO)	Rapidly optimize the parameters of extrusion 3D bioprinting (such as temperature, pressure, and speed)	Direct Ink Writing (DIW)	[138]
Artificial Neural Network (ANN)	Predicting rheological properties	Digital Light Processing (DLP)	[156]
Graph Neural Networks (GNN)	Design ink from the source of molecular structure, unlock a novel material system	Digital Light Processing (DLP)	[157]
Supported Vector Machines (SVM)	predicted the gel weight error, surface area error, and topographical heterogeneity	Direct Ink Writing (DIW)	[158]
Deep learning (DL)	Rheological properties and composition prediction	Stereolithography (SLA)	[58]
Decision Tree (DT), Random Forest (RF), Deep Learning (DL)	Printability prediction for bioinks	Direct Ink Writing (DIW)	[89]
Polynomial Fit (PF), Decision Tree (DT), Random Forest (RF)	Rheology and viscosity prediction	Direct Ink Writing (DIW)	[145]
Ridge regression (RR), K-nearest neighbor (KNN), Random Forest (RF), Neural Network (NN)	Regression/classification prediction	Stereolithography (SLA)	[159]
Random Forest (RF), LASSO, Extreme Gradient Boosting (XGBoost), Support Vector Regression (SVR)	Predicted against actual droplet velocity and volume	Ink Jetting	[160]
Random Forest (RF), Artificial Neural Network (ANN), Support Vector Machine (SVM)	To predict printability and drug dose	Ink Jetting	[161]

## Data Availability

Data sharing is not applicable to this article as no new data were created or analyzed in this study.
